# Data and its role in reducing the risk of disasters in the built environment

**DOI:** 10.1007/s11069-022-05590-7

**Published:** 2022-09-05

**Authors:** Emily So

**Affiliations:** Centre for Risk in the Built Environment, University of Cambridge, Cambridge, UK

**Keywords:** Disaster risk reduction (DRR), Data, Exposure, Vulnerability

## Abstract

In accordance with the Sendai framework, we need to protect our global neighbours from the risk of future disasters. This short paper calls for the disaster risk reduction (DRR) community to make investments in standardising and collecting the muchneeded vulnerability-relevant exposure data to help quantify, and therefore reduce risk.

The Sendai Framework for Disaster Risk Reduction 2015–2030 (Sendai Framework) was the first major agreement of the post-2015 development agenda and provides the Member States with tangible actions to protect development gains from the risk of disaster. It was endorsed by the UN General Assembly following the 2015 Third UN World Conference on Disaster Risk Reduction (WCDRR) in Sendai, Japan, and advocates for:

The substantial reduction of disaster risk and losses in lives, livelihoods and health and in the economic, physical, social, cultural and environmental assets of persons, businesses, communities and countries.

It sets as one of its priorities an ambition to realise “an understanding of disaster risk in all its dimensions of vulnerability, capacity, exposure of persons and assets, hazard characteristics and the environment”.

The equation *Risk* = *Hazard × Vulnerability × Exposure* is one that is familiar to many. Measures to understand the hazards and reduce vulnerability and exposure are therefore key to disaster risk reduction. While the scientific community is making headway in studying and monitoring different natural hazards around the world, research in quantifying exposure and defining vulnerability is still relatively underdeveloped.

Exposure is everything exposed to the risk: typically buildings and infrastructure along with the people who inhabit the buildings, itemised in terms of all those parameters that influence the vulnerability to damage from each relevant peril: such as building age, size, height, construction materials, engineered, compliant with a building code, and value.

Vulnerability-relevant exposure data, collected across a whole city, province or territory, reveals the underlying potential of buildings to be damaged by wind, earthquake shaking or flood. Defining structural vulnerability is, at best, a rather imprecise matter. The structure type is an important indicator of likely vulnerability, but the behaviour of even similar buildings will vary apparently randomly, even when subjected to the same level of threat. For example, for earthquakes, matters such as the duration and frequency content of the ground motion, the nature of the subsoil and aspects of the construction quality, including the foundations, will all have an effect that is only partly predictable.

Globalisation and rapid urbanisation have changed not only the physical landscape of most countries but have also introduced new working and living patterns. The recent COVID-19 pandemic has highlighted the fragilities and the interdependent nature of modern society. The housing of the most socially vulnerable in informal slums has been identified as physically vulnerable in earthquake risk reduction programmes for some time, but overcrowded workplaces (such as factories and food-processing plants) are not recognised as particularly vulnerable buildings. In some countries, millions of people work for long hours in proximity to substandard buildings and conditions. The non-residential sector is disparate and is not yet collectively mapped nor well understood. Multiple and parallel failures of these densely populated buildings would significantly burden search and rescue and emergency services and result in short and long-term social and economic consequences as well. Research in quantifying assets and risk in these sectors is urgently needed.

In many areas around the world, we simply do not know how much of an urban (let alone rural) environment is at risk. There have been global efforts in the earthquake engineering community, for example, to quantify global exposure. The Global Earthquake Model (GEM) Exposure Database, GED (Gamba et al. 2012), which was primarily based on the US Geological Survey’s EXPOCAT (Allen et al. 2009) and global building inventory database (Jaiswal et al. 2010), is one such initiative. The database was built to contain information on buildings and people from a country-level, down to an individual building. The first version of the GED contained aggregate information on population and the number/built area/reconstruction cost of residential and non-residential buildings at a 1 km resolution. Detailed datasets on single buildings were available for a selected number of areas, dependent on regional studies attained at the time, and it was hoped that the numbers in the repository would increase over time. However, as with most academic projects, even with GEM as custodian of the database, the expected contributions from governments, insurance companies, engineers, researchers, other organisations and individuals working in the field have not materialised, and therefore, the dataset remains stagnant and increasingly out of date.

Complementing exposure data are vulnerability functions that would estimate the likely damage to different built assets at certain levels of threat. The lack of high-quality data, both analytical and empirical, for function validation and development has been the key issue here. As with exposure data, there are many disaster-prone countries of the world that do not have vulnerability functions for many hazards and asset types.

There are therefore still far too many unknowns to realise a comprehensive understanding of global disaster risk. As advocates of the disaster risk reduction community, there are several steps one could consider:

1. Agree on a standardised set of vulnerability-relevant exposure data for different natural perils, and make a concerted effort to acquire knowledge of the key factors that affect building vulnerability in different territories in the world. Exposure data is often held in private and public repositories. The lack of transparency and standardisation on what and how data is collected is hampering progress in quantifying risks.
2. Develop innovative approaches to collect asset data at a local and global scale. For example, examining machine learning methods to identify from satellite imagery, allied with digital ground data, those parameters of the exposure that will determine, or infer, each building's vulnerability to the relevant perils at that location. This could be made publicly available and provide a global benchmark for scrutiny.
3. Agree on vulnerability function development methodologies and global data-sharing practices.

As a starting point, the Centre for Risk in the Building Environment (CURBE) and AI for the study of Environmental Risks (AI4ER) at the University of Cambridge, Risk Management Solutions (RMS) and the German Aerospace Agency (DLR) are jointly invested in a project to explore the possibility of developing synchronous, low-latency 'exposure' data, capturing total building and human exposure for a short time window. The country or countries, selected for the main phase of the research (likely to be in the Caribbean) will be locations with an elevated earthquake, hurricane or flood hazard for which we can access high-resolution imagery and also determine those parameters that will be most relevant to measuring the disaster risk. We would then measure the disaster risk associated with this exposure using a scenario reconstruction or probabilistic catastrophe loss model.

At present, we have to wait for a disaster to happen to discover what progress a country or city is making in disaster risk reduction. In the same way that insurers use probabilistic catastrophe models, with their long-term synthetic histories, to measure the annualised risk-cost, we should be able to use the same procedures to measure the risk of loss of life and building/infrastructure damage in disasters, repeated perhaps every five years to evaluate how disaster risk is changing. In effect, a risk audit.

Have the number of buildings increased even while their contribution to the overall risk has reduced because they are less vulnerable than the buildings they replaced? How has the risk landscape at a building, neighbour, city and country scale changed as a result of resilience programmes since the advent of Sendai?

Success in this project could lead to the same procedures being applied to many other territories, into a global risk audit, measuring disaster risk profiles and changes over time to assess whether countries are making progress in disaster risk reduction.

In accordance with the Sendai framework, we need to protect our global neighbours from the risk of future disasters. We can only advance our defence against the threats of natural hazards, ever more compounded by climate change, by making investments in standardising and collecting the much-needed data to help quantify risk.
